# Duodenal microbiota profiling and its effects on gastrointestinal tract dysfunction

**DOI:** 10.3389/fcimb.2026.1761015

**Published:** 2026-03-27

**Authors:** Amir Sohrabi, Fatemeh Sadeghi, Ulrika Zagai, Anna Andreasson, Michael Vieth, Lars Agréus, Nicholas J. Talley, Weimin Ye

**Affiliations:** 1 Department of Medical Epidemiology and Biostatistics, Karolinska Institutet, Stockholm, Sweden; 2 Division of Psychobiology and Epidemiology, Department of Psychology, Stockholm University, Stockholm, Sweden; 3 Department of Clinical Neuroscience, Karolinska Institutet, Stockholm, Sweden; 4 Institute of Pathology, Friedrich-Alexander-Universität Erlangen-Nürnberg, Klinikum Bayreuth, Bayreuth, Germany; 5 Bavarian Cancer Research Center (BZKF), Bayreuth, Germany; 6 Division of Family Medicine and Primary Care, Karolinska Institutet, Stockholm, Sweden; 7 School of Medicine and Public Health, University of Newcastle, New Lambton, Newcastle, NSW, Australia; 8 School of Public Health, Fujian Medical University, Fuzhou, China

**Keywords:** 16S rRNA, duodenum, gastrointestinal dysfunction, microbiota, Sweden

## Abstract

**Background:**

Duodenal microbiota has been proposed to be associated with gastrointestinal dysfunction, but population-based data are sparse. Profiling duodenal microbiota using 16S rRNA approach would appear to be a powerful tool for better understanding its role in gastrointestinal manifestations.

**Methods:**

In a population-based cross-sectional study, 265 adult subjects chosen randomly underwent symptom assessment, upper endoscopy, and gastroduodenal biopsies, with collection of duodenal brushing specimens. The 16S rRNA gene (V3-V4 region) sequencing was conducted using Illumina^©^ MiSeq platform. The microbiome taxonomy was constructed and classified to identify the microbiota composition. The diversity and composition were compared among subjects categorized based on gastrointestinal dysfunction, histopathological features, and demographic characteristics.

**Results:**

The five most abundant genera in individuals with a normal duodenum were *Streptococcus (33%), Veillonella (12%), Prevotella (11%), Rothia (5%)*, and *Actinomyces (5%).* Alpha diversity metrics showed that there were no significant differences among the participants with different demographic or histopathological features. However, the beta diversity of the duodenal microbiota differed significantly between current smokers and non-smokers, and across education level, BMI, as well as age groups. Furthermore, alteration of duodenal microbiota diversity was strongly associated with the presence of non-*H. pylori* gastritis or the co-occurrence of gastroesophageal reflux and functional dyspepsia based on Adonis R² (PERMANOVA) test (*P* < 0.05). Differential abundance of duodenal microbiota composition analysis at genus level illustrated that known pathogens and commensal bacteria, such as *Sphingomonas, Lactobacillus*, *Streptococcus, Sphingomonas, Neisseria*, *Veillonella*, *Staphylococcus, Haemophilus*, *Gemellacea*, and *Intrasporangiaceae*, were related to different histopathological manifestations.

**Conclusion:**

Alterations of duodenal microbiota signatures are linked to smoking, aging, BMI, education and gastroduodenal disorders. Further mechanistic studies are warranted to further explore the potential effects of duodenal microbiota on gastrointestinal health.

## 1 Introduction

The duodenum is located in the initial 26 cm of small intestine where nutritional absorption commences. The diversity of microbial community in the duodenum is affected by various factors, including host genetic background, diet, other lifestyle factors, and environmental influences (Cervantes et al., 2021). In addition, long-term intake of antibiotics and proton pump inhibitors (PPIs) can disrupt the balance of the microbiota in the duodenum, leading to dysbiosis. The bacterial abundance of the normal duodenum is around 10^3^ cells/g and it is taxonomically similar to the oral microbiota (Dreskin et al., 2021; Fan et al., 2021). The duodenal mucosa-associated microbiome (d-MAM) is less diverse, although it is more dynamic (Zhong et al., 2016; Cervantes et al., 2021; Dreskin et al., 2021; Fan et al., 2021). The duodenal bacteria have begun to be characterized but the fungal and viral communities in the duodenum have been less studied (Baohong et al., 2017; Gomaa, 2020; Dreskin et al., 2021; Shanahan et al., 2023). Gastroduodenal disorders can result from infections, irritants, autoimmune disorders, psychological comorbidities, and so forth. Microbial infections such as *H. pylori* are major causes of gastritis and can lead to *Helicobacter* duodenitis and duodenal ulcers. Histopathological changes in the duodenal bulb play a critical role in shaping the microbial diversity of the gastrointestinal tract.

Gastroesophageal reflux disease (GERD) and functional dyspepsia (FD) are common overlapping disorders affecting quality of life and affect up to 20% of the general population (Ronkainen et al., 2019; Jones et al., 2022).

GERD may result in reflux esophagitis or may manifest as a non-erosive reflux disease with or without histopathological changes. Meanwhile, FD is defined by the *ROME IV* classification as the presence of one or more of the following symptom clusters: postprandial fullness, early satiation, epigastric pain or epigastric burning, with no evidence of structural disease (including at upper endoscopy) that is likely to explain the manifestations. Dysbiosis and small intestinal bacterial overgrowth (SIBO) have been reported to be associated with FD in case-control studies but whether this applies in population-based outcomes, which are subject to little selection, or referral bias is uncertain. The role of the duodenal microbiome in GERD is still largely unknown and under investigation. GERD and FD are typically considered distinct diseases, although it has been consistently demonstrated that they overlap in more than 60% of cases. While the role of the microbiota in this connection remains poorly understood (Zhong et al., 2016; Andreasson et al., 2021; Hoedt et al., 2021; Klausen et al., 2021; Shah et al., 2022; Shanahan et al., 2023), it is hypothesized that the microbiota signature in FD may resemble that of GERD when compared to healthy controls.

Difficulties in sampling the duodenum segment is one of the main barriers to understand the microbiome in this region. Various specimen-collecting methods, quality of laboratory and bioinformatics analysis, participants’ characteristics and environmental contamination to enrich or impair the bacterial community are obstacles. The high throughput 16S rRNA sequencing approach is a powerful detection tool to amplify the abundance of microaerophilic, facultative anaerobic and unknown microorganisms. Scientific evidence indicates that phyla Firmicutes, Proteobacteria and Actinobacteria are dominant bacteria in the small bowel. *Streptococcus* isolates are primarily linked to gastroduodenal disorders (Qin et al., 2010; Baohong et al., 2017; Zhang et al., 2017; Quigley, 2019; Gomaa, 2020; Klausen et al., 2021; Quach et al., 2022; Shanahan et al., 2023).

However, the taxonomic classifications have presented contradictory microbiota composition across studies due to technical challenges (Qin et al., 2010; Morgan and Huttenhower, 2012; Baohong et al., 2017; Dreskin et al., 2021; Sohrabi et al., 2021; Zheng et al., 2021; Nearing et al., 2022).

Findings of large population-based studies are essential to endorse observations of the role of bacterial factors in human digestive diseases. In the current unique population-based cross-sectional study, duodenal brushing samples collected from randomly selected subjects dwelling in a Swedish community were used to characterize duodenal microbiota and its relationship to gastroduodenal disorders, as well as to study factors influencing its composition.

## 2 Methods

### 2.1 Study design

**Figure 1:**
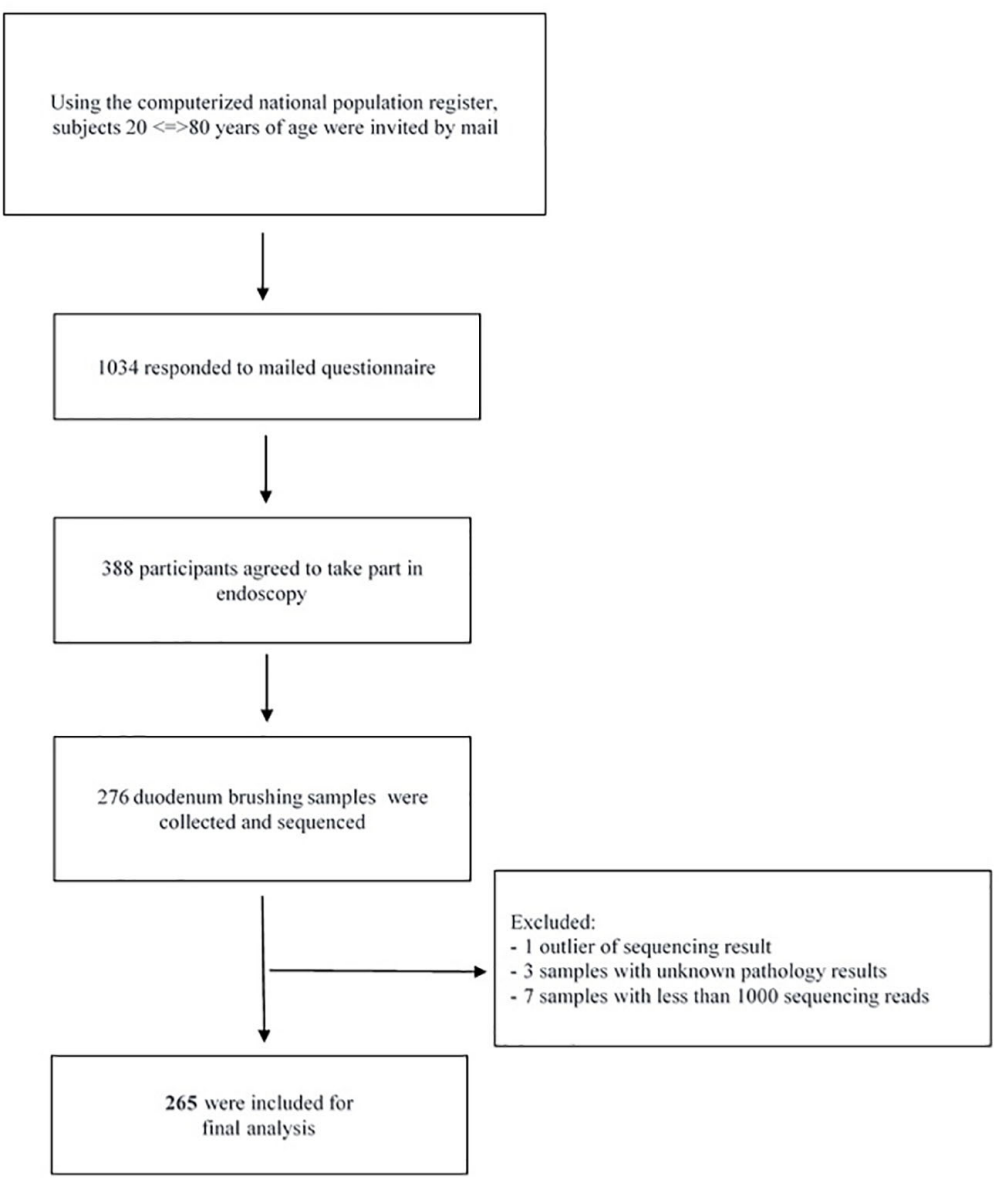
Flowchart diagram of inclusion of study population and specimens.

The current study is based on the LongGERD project, a longitudinal population-based study for detection of gastrointestinal symptoms, which was initiated in Sweden in 1989. The details of outcomes, participants’ overviews, inclusion and exclusion criteria have been described in previous reports (Agréus et al., 1995, 2001, 2016; Agreus and Simrén, 2017; Ndegwa et al., 2020). A follow-up survey was conducted in Östhammar municipality, Sweden, between January 2012 to April 2012. Among 1,842 randomly identified eligible 20–80 year old subjects (with an average age of 54 years) who answered questionnaires, 388 subjects (203 women and 185 men) underwent upper endoscopy with gastrointestinal samples taken for histopathological examination (**Figure 1**). The subjects included were representative of the overall study cohort which in turn was representative of the Swedish population. For 276 subjects, duodenum brushing samples were also taken and stored at -80°C until analysis. The study was approved by the ethics committee of the Uppsala University (Dnr 2010/443).

### 2.2 Grouping of participants

The subjects were categorized by five criteria as follows: 1- GI syndromes, functional dyspepsia (FD) and FD subgroups (postprandial distress syndrome (PDS) and epigastric pain syndrome (EPS)) based on Rome IV criteria and gastroesophageal reflux symptom (GERS) based on heartburn once a week or more often, 2- duodenum disorders, definite celiac disease, duodenal intraepithelial T lymphocytes [T-IELs] value ≥ 25/100 enterocytes, and duodenal eosinophils value ≥ 20/HPF, which was considered abnormal based on a previous population-based study (Powell et al., 2010), 3- stomach disorders and *H. pylori* status (non-atrophic *HP* gastritis, atrophic corpus *HP* gastritis, antral chemical reactive gastritis, post *HP* and seropositive, *HP* histology positive, *HP* serology positive), 4- intestinal metaplasia in the stomach, and 5- esophageal disorders objectively confirmed (esophagitis, Barrett’s esophagus, and GERS). The grouping details of the study population are listed in **Table 1**.

**Table 1:** The characteristics of the study participants.

Demographic Information	N (%)	GI Symptoms & Histopathological features	N (%)
**N**	**265**	**N**	**265**
**Sex**		**GI symptoms**	
Female	131 (49.4)	Healthy individuals (control/reference group)*	53 (20)
Male	133 (50.1)	PDS only	61 (23)
**Age**		EPS only	29 (10.9)
20-29	24 (9.1)	PDS & EPS	11 (4.2)
30-39	27 (10.2)	GERS only	54 (20.4)
40-49	49 (18.5)	GERS & Dyspepsia	29 (10.9)
50-59	54 (20.4)	**Duodenum disorders**	
60-69	82 (30.9)	Healthy individuals (control/reference group)*	53 (20)
>70	28 (10.6)	Celiac Disease	4 (1.5)
**BMI**		IEL	
Underweight-Normal (<25)	109 (41.1)	<25	256 (96.6)
Overweight (25-29.9)	111 (41.9)	>=25	8 (3)
Obesity (>=30)	38 (14.3)	Eosinophils	
**Education**		<20	262 (98.8)
<Upper Secondary School	84 (31.7)	>=20	3 (1.2)
>=Upper Secondary School	175 (66.1)	Dyspepsia & Eosinophils	3 (1.2)
**Current smoking**		**Stomach disorders & *H. pylori* status**	
No	228 (86)	Healthy individuals (control/reference group)*	53 (20)
Yes	35 (12.9)	Non-atrophic HP gastritis	22 (8.3)
**Current snuff dipping**		Atrophic corpus HP gastritis	7 (2.6)
No	226 (85.3)	Antral chemical reactive gastritis	48 (18.1)
Yes	37 (13.9)	Post *H. pylori* and sero positive	19 (7.1)
**Current alcohol consumption**		HP histology positive	28 (10.6)
No	47 (17.8)	HP serology positive	34 (12.8)
Yes	216 (81.5)	**Intestinal metaplasia**	
**PPI/H2 intake (last week)**		Healthy individuals (control/reference group)*	53 (20)
No	243 (91.7)	Yes	9 (3.4)
Yes	19 (7.2)	**Esophageal disorders**	
**PPI/H2 intake (<3 months)**		Healthy individuals (control/reference group)*	53 (20)
No	229 (86.4)	Esophagitis only	24 (9)
Yes	33 (12.5)	GERS only	49 (18.5)
		Barrett’s esophagus only	20 (7.6)
		Esophagitis & GERS	14 (5.3)
		Barrett’s esophagus & GERS	3 (1.1)
		Esophagitis & Barrett’s esophagus	1 (0.4)
		Esophagitis & Barrett’s esophagus & GERS	3 (1.1)

BMI, Body Mass Index (BMI was calculated as weight (kg)/(height m^2^); PPI, Proton Pump Inhibitor (PPI/H2 beta blockers intake is any time during last week & last 3 months); PDS, Postprandial Distress Syndrome; EPS, Epigastric Pain Syndrome; IEL, intraepithelial T lymphocytes; HP, *H. pylori*; GERS, Gastroesophageal Reflux Symptom. Education was classified as less than upper secondary school, or upper secondary school, and higher.* Healthy individuals (control/reference group) means asymptomatic and non-pathological manifestations of any gastrointestinal disorders.

### 2.3 Bacterial 16S rRNA sequencing workflow

The DNAs of duodenum brushing samples were extracted using Mag Maxi kit (LGC group^©^, Germany). Briefly, two-step nested PCR amplification procedures were carried out using forward (341) and reverse (805) primers (Ndegwa et al., 2020). During the first and second steps of nested PCR, the V3-V4 regions of the bacterial 16S rRNA were amplified, targeted, and indexed using barcoded Illumina^©^ adapters and linkers. A Mock microbial community’s standard and home-brew positive controls (*H. pylori* HPAG1 and DU30 strains plus *Lactobacillus*), as well as non-template controls, were added during the library preparation process for quality control assessment. Amplicons were generated using KAPA Hifi HotStart ReadyMix (2X) (Roche^©^, Germany) in a 50 µL reaction volume. Afterwards, they were purified using 1.8 x Agencourt AMPure XP (Beckman Coulter, Inc.). Libraries were pooled and adjusted to 4nM using Qubit^®^ 2.0 Fluorometer (Invitrogen^©^) and the average fragment length of 600 bp was identified for the final library. The spiked library was submitted to SciLife-Lab (science for life laboratory/national genomics infrastructure), Stockholm, Sweden for sequencing. The Illumina^©^ Miseq platform was used for 16S rRNA sequencing on 10pM library and 10% PhiX using 2 ×300 bp paired-end protocol of Miseq V3 reagents (Illumina Co.).

### 2.4 Bacterial 16S rRNA bioinformatics analysis

Sequencing of bacterial 16S rRNA gene amplicons from the duodenal microbiota generated FASTQ-format files containing raw sequence reads for targeted microbiome analysis. DNA demultiplexed paired sequence reads were imported into the QIIME 2 in a Python 3.8 version (Bolyen et al., 2019). The quality score of 30 (Q30 is equal to 1 in 1000 probability of incorrect base call) was considered to clean and trim the nucleotides fragments during DADA2 de-noising process by removing specific assigned barcodes-linkers, PhiX, background noisy reads and repetitions from individual samples. The poor-quality reads with more than 2 expected errors were filtered and discarded from the analysis. The operational taxonomic units (OTUs) were generated and were aligned to the Greengenes database (version 13.5) to develop a quantitative strategy based on observed 99% similarities for classifying organisms into groups (DeSantis et al., 2006).

For diversity analysis, samples were rarefied to 3000 reads. Alpha diversity indices (Shannon index, Evenness index, and Observed OTUs) and beta diversity metrics (weighted UniFrac, unweighted UniFrac, Jaccard, and Bray-Curtis metrics) were calculated on rarefied data using QIIME2. The Beta diversity was analyzed and compared using Adonis: permutational multivariate test of variance (PERMANOVA) through R vegan library (v 2.5-2). The analyses were adjusted for age (20-29, 30-39, 40-49, 50-59, 60-69, and ≥70), BMI (≤25, 25-29.9, ≥30), education (less than upper secondary school, or upper secondary school, and higher), sex (male, female), and current smoking (no, yes) with 9,999 permutations. The factors affecting duodenal microbiota have not been fully established. Thus, the significant factors in univariate analysis were considered as confounding factors in the model (Anderson, 2001). The *Principal Coordinate Analysis* (PCoA) was also performed on beta diversity indices using R, and the outputs were visualized using ggplot2.

Microbiome differential abundance analysis was conducted to identify individual taxa of which relative abundances were significantly different among various groups using DESeq2, a negative binomial distribution model in R environment (R Core Team, 2013), while adjusting for potential confounding factors such as age, sex, BMI, proton pump inhibitors (PPIs)/H2-blocker intake and current smoking. The coefficient factor with -2.5 ≥ log2 fold change ≥ 2.5 (FDR-adjusted p <0.05) was applied for considering the bacterial species that at least 5 times less or 5 times more associated with GI symptoms, and histopathological manifestations.

## 3 Results

Out of 276 sequenced duodenal specimens, 1 outlier sequence, 3 samples with unknown pathology results and 7 samples with less than 1000 sequence reads were discarded from the current analysis process. Therefore, 265 sequenced duodenum samples with an average of 36,763 reads per sample were chosen for further analysis. Non-chimeric reads were clustered at 99% identification into 2,682 OTUs. In addition, features less than 2% of the total samples were removed, so the 971 OTUs were selected for final analysis (**Figure 1**).

The 265 individuals aged 20–79 years were included in the assessment (49.4% female *vs* 50.1% male, mean age: 53.2 **±** 14.4) (**Table 1**). In GI symptom groupings, 11 (4.2%) had functional dyspepsia (postprandial distress syndrome (PDS) or epigastric pain syndrome (EPS)), and 29 (10.9%) individuals had GERS and functional dyspepsia concurrently.

The histopathological features of duodenal disorders demonstrated that 3 (1.2%) cases had ≥ 20 eosinophils per HPF, 3 (1.2%) individuals had dyspepsia and eosinophils simultaneously, 8 (3%) had high intraepithelial T lymphocytes [T-IELs] greater than 25, and 4 (1.5%) had confirmed active celiac disease on histology (CD). The IEL counts were greater than 45 in all the CD subjects.

In the gastric disorders grouping, 53 subjects (20%) were considered as healthy individuals (asymptomatic and non-pathological manifestations of any gastrointestinal disorders). Thirty-four cases (12.8%) were seropositive for past *H. pylori* infection and 28 cases (10.6%) were currently infected with *H. pylori.* The prevalence rates of non-atrophic *HP* gastritis, atrophic corpus *HP* gastritis, and antral chemical reactive gastritis were 8.3%, 2.6%, and 18.1%, respectively. In addition, 9 (3.4%) participants suffered from intestinal metaplasia.

In the esophageal disorders grouping, 49 (18.5%) subjects had only GERS, while 24 (9%) and 20 (7.6%) subjects had esophagitis and Barrett’s esophagus, respectively (**Table 1**).

### 3.1 16S rRNA sequencing

**Figure 2:**
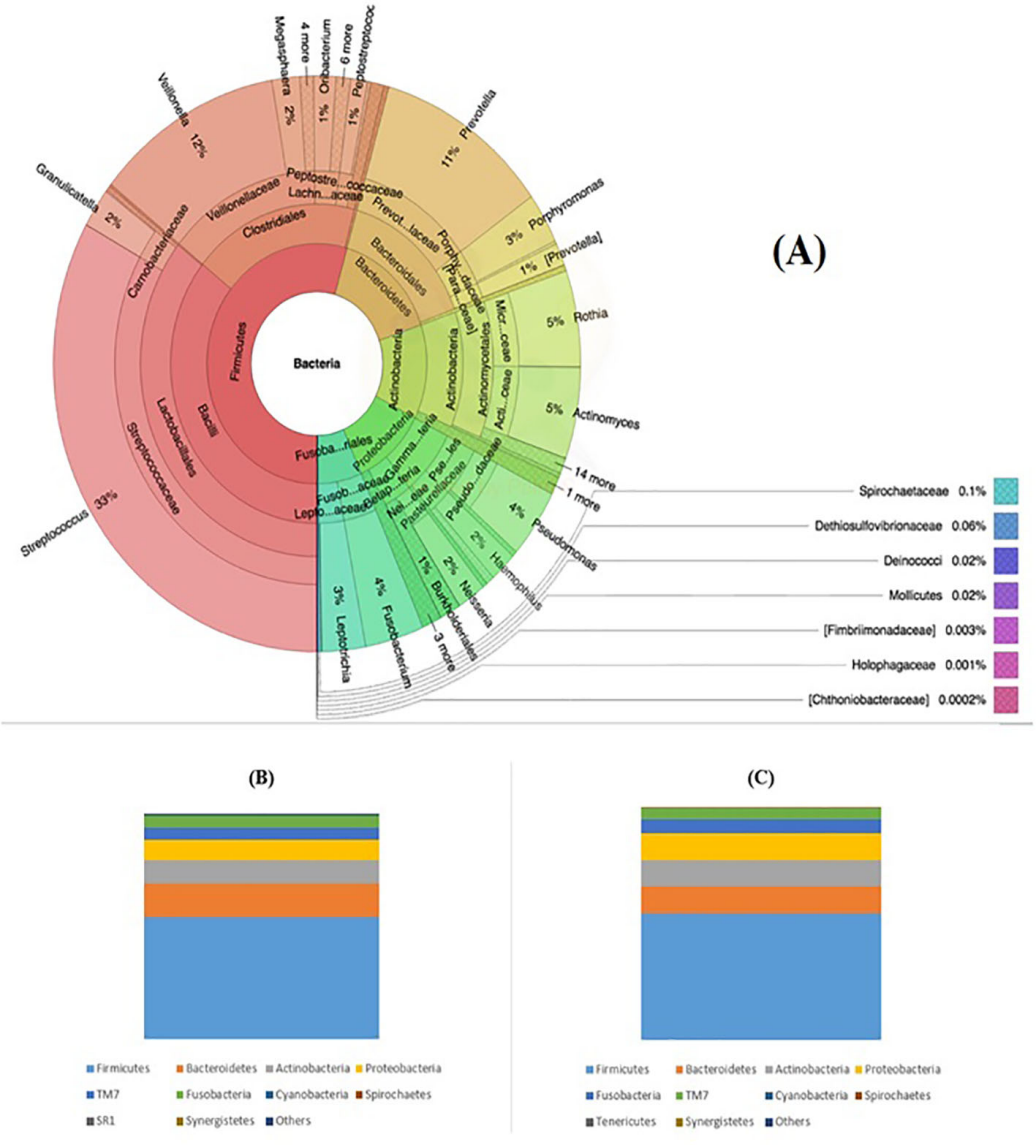
Taxonomic composition of the duodenal microbiota. **(A)** Bacterial composition of the normal duodenum visualized by a Krona plot. The concentric circles represent taxonomic levels from kingdom (inner circle) to genus (outer circle); **(B)** Relative abundance of the major bacterial phyla in individuals with a normal duodenum; and **(C)** Relative abundance of the dominant bacterial phyla in participants with duodenal disorders.

Microbial taxonomy analysis identified 18 bacterial phyla in subjects with normal duodenum. Top phyla were Firmicutes (54%), Bacteroidetes (15%), Actinobacteria (13%), Proteobacteria (11%), and Fusobacteria (6%). At the genus level, the five dominant genera were *Streptococcus* (33%), *Veillonella* (12%), *Prevotella* (11%), *Rothia* (5%), and *Actinomyces* (5%). In addition, top phyla detected in the normal and abnormal duodenal individuals are shown in **Figure 2**.

### 3.2 Duodenal microbiota diversity indices

Analysis of intra-sample (alpha) diversity metrics demonstrated that microbial richness and evenness of the duodenal microbiota did not differ significantly across demographic characteristics, gastrointestinal (GI) symptom categories, or histopathological features (all P > 0.05; data not shown). These findings suggest that, despite clinical and demographic heterogeneity, the overall within-sample diversity of the duodenal microbiota remained relatively stable among the studied outcomes.

**Figure 3:**
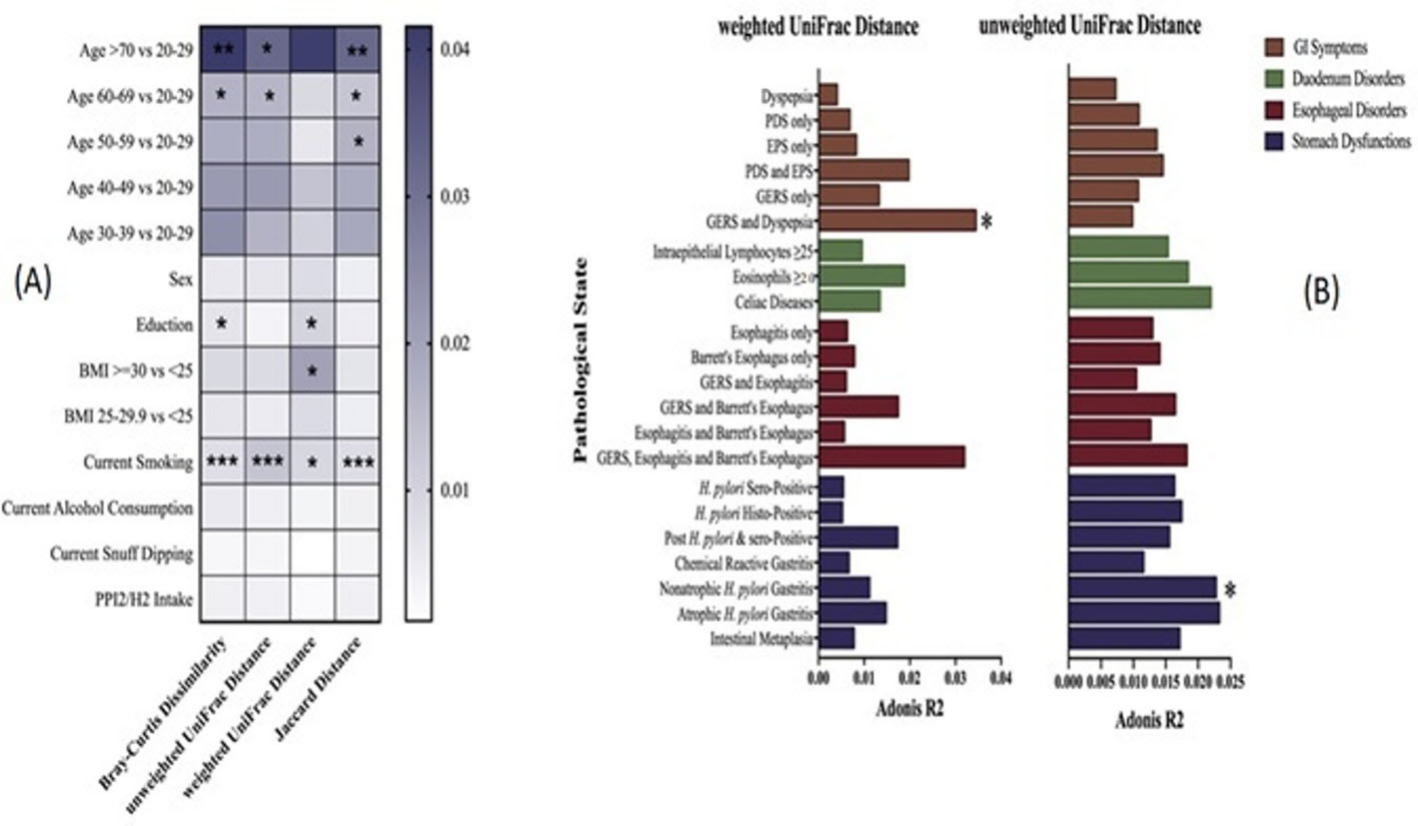
Association between beta diversity and demographic, clinical, and pathological variables assessed by Adonis (PERMANOVA). **(A)** Adonis R² values for associations between demographic factors and microbial beta diversity measured using Bray-Curtis dissimilarity, unweighted UniFrac distance, weighted UniFrac distance, and Jaccard distance; and **(B)** Adonis R² values for associations between gastrointestinal symptoms and histopathological features with microbial beta diversity based on weighted and unweighted UniFrac distances, adjusted for age group, BMI, education level, and current smoking status (9,999 permutations). P-value significance levels are indicated (*P < 0.05, **P < 0.01).

In contrast, between-sample (beta) diversity analyses revealed significant differences in microbial community composition across several demographic and clinical variables. As summarized in **Figure 3A**, Adonis (PERMANOVA) models demonstrated that age group, education level, body mass index (BMI), and current smoking status were significantly associated with variation in duodenal microbial beta diversity when assessed using Bray-Curtis dissimilarity, unweighted UniFrac distance, weighted UniFrac distance, and Jaccard distance metrics (P < 0.05 and P < 0.01). These associations were consistently observed across multiple distance measures, indicating that both taxonomic composition and phylogenetic structure of the duodenal microbiota varied according to these demographic and lifestyle factors. Further Adonis analyses focusing on clinical and pathological variables identified additional associations with microbial beta diversity after adjustment for age group, BMI, education level, and smoking status (**Figure 3B**). Specifically, individuals with non-atrophic *Helicobacter pylori* gastritis and those presenting with both gastroesophageal reflux symptoms (GERS) and dyspepsia exhibited significant differences in microbial community structure compared with healthy controls, based on both weighted and unweighted UniFrac distances (P < 0.05). These findings suggest that disease-associated differences in the duodenal microbiota involve shifts in both the presence or absence of taxa and their relative abundances.

**Figure 4:**
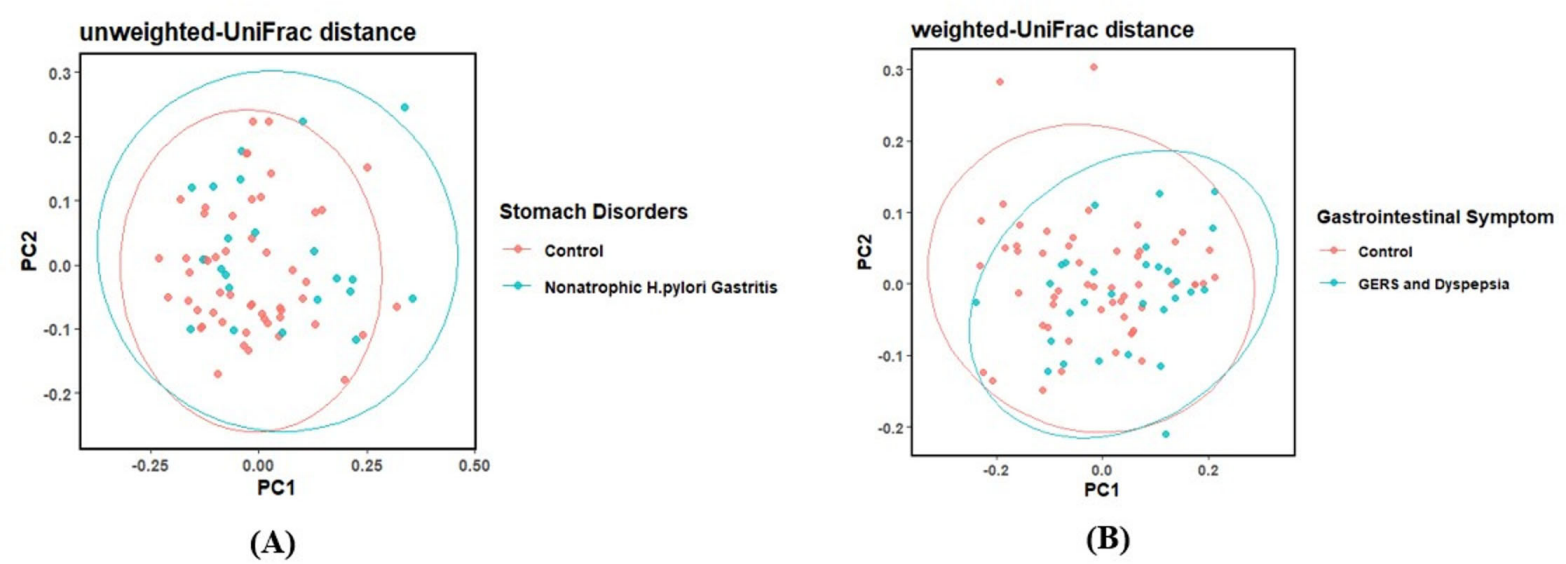
Principal Coordinates Analysis (PCoA) of microbial beta diversity for groups showing significant differences in Adonis models. PCoA plots based on UniFrac distances visualize group separations for variables that were significantly associated with microbial community structure in Adonis analyses (P < 0.05). **(A)** Stomach disorder groups; and **(B)** Gastrointestinal symptom groups.

To visualize these statistically significant beta-diversity differences, principal coordinates analysis (PCoA) plots were generated for the relevant comparison groups (**Figure 4**). As shown in **Figure 4A**, PCoA based on UniFrac distances revealed partial separation between individuals with non-atrophic *H. pylori* gastritis and healthy controls, indicating differences in overall microbial community structure associated with stomach disorders. Similarly, **Figure 4B** indicates separation between participants with combined GERS and dyspepsia and controls, supporting the Adonis findings that GI symptom status is associated with distinct duodenal microbial community patterns. Although overlaps between groups were observed, the clustering patterns demonstrate measurable shifts in microbial composition rather than complete community restructuring.

### 3.3 Duodenal microbiome composition analysis

**Figure 5:**
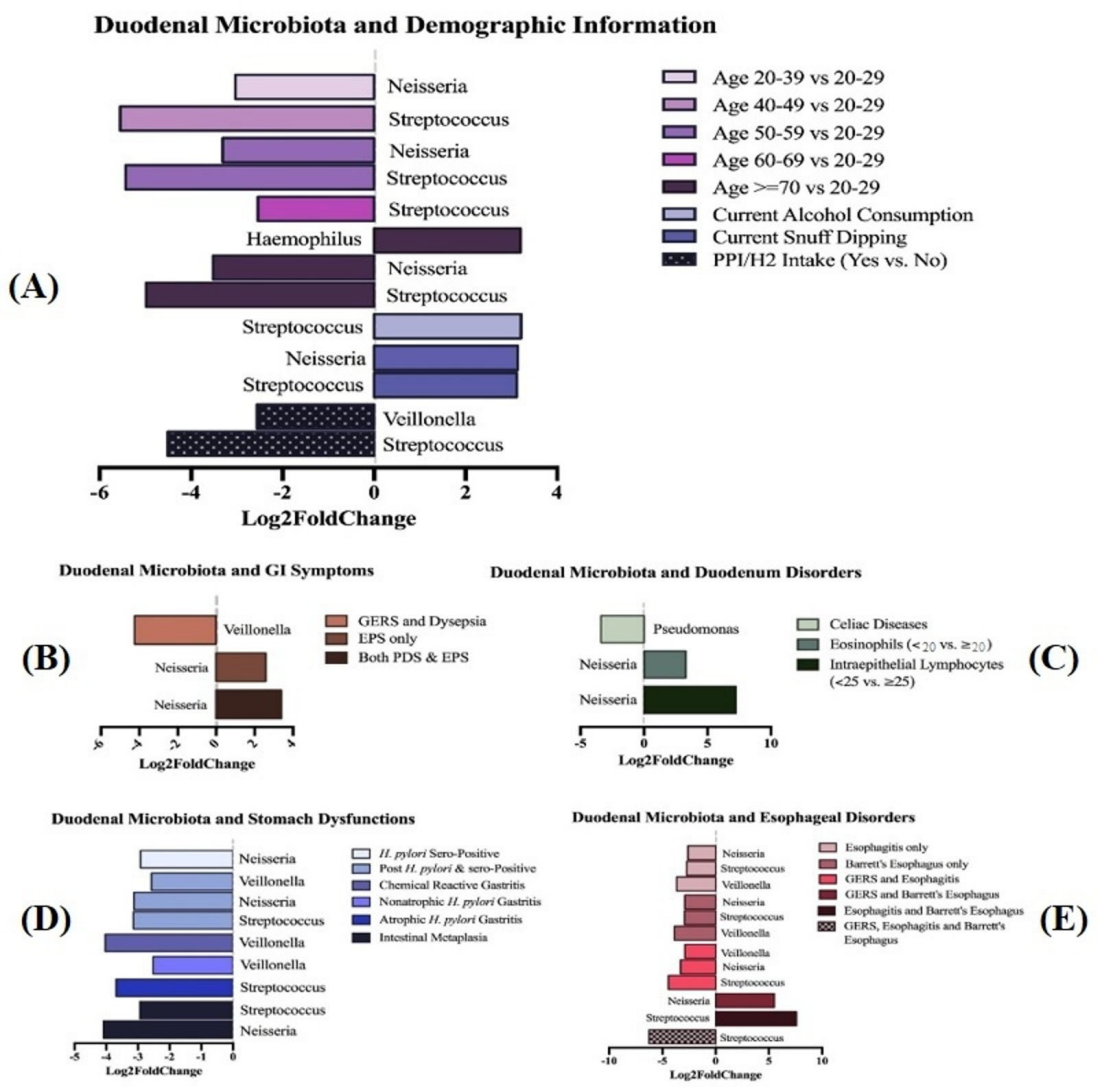
Differential taxonomy analysis of the duodenal microbiota across clinical and demographic groups using the DESeq2 method. **(A)** Demographic characteristics; **(B)** gastrointestinal (GI) symptoms; **(C)** duodenal disorders; **(D)** stomach dysfunctions; and **(E)** esophageal disorders. Only bacterial genera with an absolute log2 fold change ≥ 2.5 and a FDR-adjusted P-value < 0.05 are shown. All analyses were adjusted for age, sex, body mass index (BMI), proton pump inhibitor (PPI) or H2-blocker intake, and smoking status to control for potential confounding factors.

To identify specific bacterial taxa contributing to these observed differences, differential abundance analysis was conducted using the DESeq2 method (**Figure 5**). At the genus level, several taxa-including *Streptococcus*, *Lactobacillus*, *Sphingomonas*, *Neisseria*, *Veillonella*, *Staphylococcus*, *Haemophilus*, *Gemellaceae*, and *Intrasporangiaceae* showed significant positive or negative associations across demographic characteristics, GI symptom groups, and pathological conditions. As illustrated in **Figure 5A**, age group, alcohol consumption, snuff (snus) dipping, and PPI or H2-blocker use were associated with differential abundance of specific genera. Additional subgroup analyses demonstrated distinct microbial signatures associated with GI symptoms (**Figure 5B**), duodenal disorders (**Figure 5C**), stomach dysfunctions (**Figure 5D**), and esophageal disorders (**Figure 5E**). Only taxa meeting stringent criteria (absolute log2 fold change ≥ 2.5 and P < 0.05), after adjustment for relevant confounders, were included, highlighting robust associations between clinical manifestations and alterations in duodenal microbial composition.

## 4 Discussion

One of the primary goals of the current population-based study was to evaluate the association between duodenal microbiota profiling and upper GI diseases and disorders. Duodenal microbiota signature might reflect the dysbiosis of digestive tract (Gong et al., 2019; Han et al., 2019; Suárez-Jaramillo et al., 2020; Chung et al., 2021). It is supposed that a normal GI tract might have a different microbial diversity, richness and abundance compared to that with GI disorders. Hence, composition of microbiota might also be altered at various stages of the GI precancerous cascade. Lifestyle, mental disorders, antibiotics intake, ethnicity, and residency in developed communities are highlighted as possible factors related to microbiome diversity (Aviles-Jimenez et al., 2014; Eun et al., 2014; Giamarellos-Bourboulis et al., 2015; Li et al., 2017; Coker et al., 2018; Singh et al., 2018; Gong et al., 2019; Shi et al., 2019; Ndegwa et al., 2020; Shah et al., 2022; Shanahan et al., 2023).

In the present study, duodenal dysbiosis was characterized primarily by alterations in microbial community composition rather than changes in overall diversity or richness, with significant beta-diversity differences observed in individuals with functional dyspepsia and overlapping GERS. Our findings also suggest that the duodenal microbiota is abnormal in non-atrophic *H. pylori* gastritis, GERS, and functional dyspepsia. Notably, the microbiota in patients with both GERS and functional dyspepsia showed significant shifts compared with healthy controls, implying that alterations in the duodenal microbial community are linked to these disorders. While GERD and functional dyspepsia have traditionally been considered as separate diseases (Talley, 2013; Quach et al., 2022), however, their significant clinical overlap and the shared microbial changes observed in our study suggest a potential common microbiota-related mechanism. To our knowledge, this is the first study to evaluate the impact of having both dyspepsia and GERD together on the duodenal microbiota, providing novel insight into their possible pathophysiological links and underscoring the need for further research to better understand the clinical implications of these microbial shifts. These compositional alterations support the concept of the duodenum as a pathogenic center in symptom generation, indicating that even subtle microbial imbalances may contribute to symptoms without obvious histological abnormalities. In line with the model proposed by Miwa et al (Miwa et al., 2019), the duodenum may function as a key regulatory site where luminal factors and mucosal responses interact to drive symptom development. Miwa et al. emphasized that disturbed duodenal signaling can lead to hypersensitivity and abnormal gastric sensorimotor function. Consistent with this framework, changes in the relative abundance of specific genera in our study may influence the duodenal microenvironment, potentially promoting immune activation, low-grade inflammation, increased mucosal permeability, and heightened mucosal sensitivity. Together, these mechanisms could facilitate duodenal hypersensitivity and subsequent symptom generation. Although inflammatory markers were not directly assessed, the identified symptom-associated microbial signatures are compatible with a primed duodenum contributing to functional gastrointestinal symptoms. Clinically, these findings indicate that targeting the duodenal microenvironment and its microbiota, alongside conventional acid-suppressive therapy, may represent a complementary strategy in the management of functional dyspepsia and related reflux symptoms. The present results underscore the need for further interventional research to better understand the clinical implications of these microbial shifts.

Histopathological changes in the duodenum and bulb such as incomplete gastric metaplasia, focal inflammation, and lymphocytosis are associated with differences in microbiota composition. Therefore, non-atrophic *Helicobacter pylori* gastritis is expected to exhibit a distinct microbial diversity compared to other types of gastritis.

Characteristics and potential risk factors, including lifestyle, aging, smoking, snuff (snus) dipping, alcohol consumption, low education level, BMI, intake of PPIs/H2 and beta blockers have been appraised to have significant effects on the bacterial composition of digestive tract, particularly duodenal microbiota (Aviles-Jimenez et al., 2014; Eun et al., 2014; Giamarellos-Bourboulis et al., 2015; Li et al., 2017; Coker et al., 2018; Singh et al., 2018; Shi et al., 2019; Zheng et al., 2021). Specifically, microbial composition of the digestive tract is gradually altered in the aging process. Our results demonstrated that microbiota features of age groups ≥40 years were significantly different from those <40 years, which were similar to previous scientific reports (O’Toole and Jeffery, 2015; Song et al., 2015; Kim and Benayoun, 2020). Furthermore, in current smokers (35, 12.9%) compared to non-smokers (228, 86%), there was a significant difference in microbiota abundance, phylogeny distance, and distribution. The study of Shanahan et al. supports our findings, showing how the diversity of duodenal microbiota is influenced by smoking as a known influential factor (Shanahan et al., 2023).

Taxonomic compositions of bacteria at the phylum level were reasonably similar to what have been shown in scientific reports of the overall composition of duodenal microbiota. However, the relative abundance of *Proteobacteria* in the present population (11%) was lower than in previous studies conducted in Italy (40%) (Nardelli et al., 2020) or the USA (14.8%) (Leite et al., 2019), which might be due to the fact that our study participants lived in a low-*H. pylori* prevalence area (Sweden) (Ndegwa et al., 2020). Mobeen et al. confirmed the five top genus-level combinations of *Streptococcus* (33%), *Veillonella* (12%), *Prevotella* (11%), *Rothia* (5%), and *Actinomyces* (5%). The ratio of gut *Proteobacteria* (P) and *Actinobacteria* (A) in all Western countries was >1, while Sweden exhibited a P/A ratio <1 (Mobeen et al., 2018).

Scientific studies of duodenal microbiota in GI disorders suggested a bacterial diversity changes in the stomach and duodenum segments, which may contribute to progression of gastric, duodenum and intestinal disorders (Quigley, 2019; Gong et al., 2019; Han et al., 2019; Cervantes et al., 2021; Chung et al., 2021; Shah et al., 2022; Shanahan et al., 2023).

​In our large general population–based cross-sectional study, four patients (1.5%) with celiac disease (CD) had elevated intraepithelial T lymphocytes (T-IELs; ≥45). However, in contrast to previous studies (Giamarellos-Bourboulis et al., 2015; Harnett et al., 2016; Singh et al., 2018; Labruna et al., 2019; Shah et al., 2022), we observed no significant association between CD and bacterial diversity, possibly due to the small sample size.

Human host factors and environmental conditions have a principal impact on balancing gut microbiota. Commensal and flora bacteria, alongside pathogenic agents, have a high potential risk for altering the microbiome profile of the gastrointestinal tract. Comparison of duodenal composition using the DESeq2 approach showed that significant changes at the genus level included *Lactobacillus*, *Streptococcus*, *Sphingomonas*, *Neisseria*, *Veillonella*, *Staphylococcus*, *Haemophilus*, *Gemellacea*, and *Intrasporangiaceae*. It appears duodenal bacterial diversity at the mucosa is influenced by medication intake, and risk factors related to demographic backgrounds as well (Peris-Bondia et al., 2011; Coker et al., 2018; Arroyo Vázquez et al., 2019; Chiang and Lin, 2019; Labruna et al., 2019; Shi et al., 2019; Fukui et al., 2020; Muhamad Rizal et al., 2020; Suárez-Jaramillo et al., 2020; Sung et al., 2020; Wauters et al., 2020; Cervantes et al., 2021).

The study strengths include inclusion of a random population sample who underwent upper endoscopy, a relatively unique population including subjects with disease and healthy controls. All subjects were carefully phenotyped, including symptom assessments, histology and endoscopy examinations. The main limitation is microbial community profiling using 16S rRNA gene sequencing rather than shotgun sequencing, as this may lead to missed or uncharacterized bacteria during data analysis. In addition, challenges in laboratory and bioinformatics workflows are other limitations (Muhamad Rizal et al., 2020; Hoedt et al., 2021). Last, human DNA contamination from the mucosa makes deeper sequencing much more challenging. More accurate and definitive duodenal microbiome signatures need to be studied to determine clinical relevancies in the future.

## 5 Conclusion

In conclusion, our findings illustrated that alterations in duodenal microbiome composition were strongly associated with non-atrophic *H. pylori* gastritis, GERS, and dyspepsia. The diversity and abundance of duodenal microbiota were also strongly associated with current smoking, and were correlated with education level, obesity (BMI ≥ 30), as well as age (≥ 40 years). Furthermore, taxonomic analysis demonstrated that known bacterial species and flora differed significantly in duodenal microbiota composition across various digestive tract disorders and demographic backgrounds. Notably, bacteria at the genus level most linked to GI symptoms and histopathological features included *Streptococcus*, *Lactobacillus*, *Sphingomonas*, *Neisseria*, *Veillonella*, *Staphylococcus*, *Haemophilus*, *Gemellacea*, and *Intrasporangiaceae*. More efforts with larger sample sizes in different ethnic communities using high-throughput improved 16S rRNA sequencing or, preferably, metagenomic sequencing approaches would help overcome the difficulties of duodenal microbiota profiling for preventing and predicting gastrointestinal diseases.

## Data Availability

The original contributions presented in the study are included in the article, further inquiries can be directed to the corresponding author.
